# Effluent-Dose Response of Continuous Haemofiltration Integrated into Veno-Venous ECMO for Septic Shock: A Retrospective Cohort Study

**DOI:** 10.3390/medicina61091653

**Published:** 2025-09-11

**Authors:** Nicoleta Barbura, Tamara Mirela Porosnicu, Cristian Oancea, Dorel Sandesc, Marius Papurica, Ovidiu Bedreag, Ciprian Gîndac, Adelina Raluca Marinescu, Ruxandra Laza, Voichita Elena Lazureanu

**Affiliations:** 1 Doctoral School, “Victor Babes” University of Medicine and Pharmacy, Eftimie Murgu Square 2, 300041 Timisoara, Romania; nicoleta.marian@umt.ro; 2 Anaesthesia and Intensive Care Research Center, Faculty of Medicine, “Victor Babes” University of Medicine and Pharmacy, Eftimie Murgu Square 2, 300041 Timisoara, Romania; sandesc.dorel@umft.ro (D.S.); marius.papurica@umft.ro (M.P.); bedreag.ovidiu@umft.ro (O.B.); ciprian.gindac@umft.ro (C.G.); 3 Center for Research and Innovation in Precision Medicine of Respiratory Diseases (CRIPMRD), “Victor Babes” University of Medicine and Pharmacy, Eftimie Murgu Square 2, 300041 Timisoara, Romania; 4 Discipline of Infectious Disease, “Victor Babes” University of Medicine and Pharmacy, Eftimie Murgu Square 2, 300041 Timisoara, Romania; adelina.marinescu@umft.ro (A.R.M.); laza.ruxandra@umft.ro (R.L.); lazureanu.voichita@umft.ro (V.E.L.)

**Keywords:** septic shock, extracorporeal membrane oxygenation, haemofiltration, renal replacement therapy, critical care outcomes

## Abstract

*Background and Objectives*: The optimal effluent dose of continuous haemofiltration (CHF) when coupled to veno-venous extracorporeal membrane oxygenation (ECMO) for septic shock is unknown. We examined our 44-patient ECMO registry, contrasting a smaller high-dose subgroup (HDHF ≥ 45 mL kg^−1^ h^−1^; n = 13) with a larger standard-dose subgroup (SDHF 25–35 mL kg^−1^ h^−1^; n = 31). The primary endpoint was 72 h change in SOFA score (ΔSOFA). *Materials and Methods*: All adults cannulated for ECMO (January 2018–January 2025) and started on CHF within 2 h were eligible. Variables were abstracted at baseline, 24 h and 72 h. Continuous data were analysed by Student’s *t* or Mann–Whitney tests, categorical data by χ^2^/Fisher; and paired changes by Wilcoxon. Two-sided *p* < 0.05 signified significance. *Results*: Baseline characteristics were comparable (age 49.1 ± 15.2 vs. 50.4 ± 14.9 y; APACHE II 28.4 ± 5.3 vs. 27.5 ± 5.9). Median effluent reached 48.1 mL kg^−1^ h^−1^ (IQR 46.6–49.7) in HDHF and 29.7 mL kg^−1^ h^−1^ (27.5–31.9) in SDHF (*p* < 0.001). IL-6 fell by 1 061 ± 487 pg mL^−1^ with HDHF versus 637 ± 425 pg mL^−1^ with SDHF (*p* = 0.003). Mean arterial pressure rose 19.2 ± 8.1 vs. 12.7 ± 8.3 mmHg (*p* = 0.03), and norepinephrine declined 0.46 ± 0.22 vs. 0.30 ± 0.19 µg kg^−1^ min^−1^ (*p* = 0.04). ΔSOFA at 72 h was –4.4 ± 2.1 with HDHF and –2.6 ± 2.3 with SDHF (*p* = 0.01). Twenty-eight-day mortality was 38.5% (5/13) versus 45.2% (14/31), *p* = 0.64. Effluent dose correlated with ΔIL-6 (ρ = 0.53, *p* < 0.001) and ΔSOFA (ρ = 0.45, *p* = 0.003). *Conclusions*: In this ECMO cohort, high-dose haemofiltration, although applied in only 13 patients, appeared to achieve greater cytokine clearance, faster haemodynamic recovery and deeper early organ-failure improvement than standard dosing, without excess bleeding. Survival advantage was not demonstrable, underscoring the need for prospective randomised confirmation of the dose–response signal.

## 1. Introduction

Septic shock is defined as life-threatening organ dysfunction caused by a dysregulated host response to infection, accompanied by persistent hypotension and hyperlactataemia despite adequate resuscitation, according to the Sepsis-3 consensus definition [1]. Current U.S. surveillance data show that sepsis complicates 6% of all adult hospitalisations—about 1.7 million cases annually—with in-hospital mortality that has plateaued near 35–40% after a decade of gradual decline [2]. The 2021 Surviving Sepsis Campaign recommends early antimicrobial therapy, haemodynamic optimisation and timely organ-support strategies, explicitly acknowledging extracorporeal techniques when conventional measures fail [3]. In parallel, use of extracorporeal membrane oxygenation (ECMO) for adult septic shock is climbing; the prospective ECMO-RESCUE cohort anticipates that veno-arterial support may improve 30-day survival in refractory septic cardiogenic shock, highlighting the growing clinical equipoise around this intervention [4].

Pathogen- and damage-associated molecular patterns trigger a cytokine storm that precipitates distributive hypotension, mitochondrial injury and the reversible biventricular depression known as sepsis-induced cardiomyopathy [5]. Among circulating mediators, interleukin-6 (IL-6) concentrations > 1000 pg mL^−1^ correlate strongly with vasopressor dependence and early mortality. Medium cut-off haemofilters such as EMiC2 achieve IL-6 sieving coefficients of 0.35–0.45 and can lower systemic levels by ≈25% within the first 24 h of treatment in septic AKI patients [6]. A comprehensive review of extracorporeal blood-purification technologies confirms that flow-dependent convective clearance—not merely membrane chemistry—is critical if immunomodulation is the therapeutic goal [7].

The appropriate “dose” of haemofiltration for immunomodulation remains contentious. In the multicentre IVOIRE randomised trial, an effluent of 70 mL kg^−1^ h^−1^ did not confer a mortality advantage over the renal-dose standard of 35 mL kg^−1^ h^−1^ but did shorten norepinephrine exposure [8]. A 2020 meta-analysis synthesising 14 studies reached a similar conclusion, citing marked heterogeneity and insufficient power to detect modest survival benefits [9]. Nevertheless, systematic reviews focused on septic AKI suggest haemodynamic gains when clearance exceeds 45 mL kg^−1^ h^−1^ [10], and two trials in burn-related septic shock demonstrated reversal of vasoplegia and organ-failure scores at effluent targets of 50–80 mL kg^−1^ h^−1^ [11,12]. Beyond sheer flow, membrane adsorptivity matters: an AN69ST surface-treated filter reduced the vasopressor-dependency index faster than a polysulfone membrane despite identical effluent rates in a 38-patient septic cohort [13].

Coupling continuous renal replacement therapy (CRRT) to the ECMO circuit is technically straightforward but alters pharmacokinetics and mediator dynamics. Downstream placement of a filtration cartridge, for example, removes pro-inflammatory cytokines but can reduce vancomycin troughs by nearly 50% if dosing is not adjusted [14]. Likewise, pathogen-binding devices such as the Seraph-100^®^ filter have shown rapid haemodynamic stabilisation and lactate clearance when used in series with ECMO during COVID-19 septic shock [15]. In contemporary veno-arterial ECMO cohorts, CRRT is favoured over peritoneal dialysis for >80% of patients; however, a 2024 comparative study reported similar in-hospital mortality between modalities while noting faster achievement of net-negative fluid balance with CRRT [16].

Large registries underscore the complexity of the ECMO-CRRT interface. Obesity increases thrombosis risk and the likelihood of CRRT initiation without independently worsening survival [17], whereas invasive fungal disease complicates ≈5% of VA-ECMO runs and carries a mortality rate of >90% [18]. After four decades of technological refinement, CRRT practice remains variable worldwide, as highlighted in recent anniversary reports and practice surveys that document wide dispersion in dose prescription, anticoagulation strategy and effluent composition despite converging guidelines [19,20]. This heterogeneity hampers meta-analytic clarity and underscores the need for sepsis-specific, dose-controlled investigations.

Against this backdrop, we performed a retrospective cohort study comparing high effluent dose and standard effluent dose continuous haemofiltration delivered through the ECMO circuit in adults with septic shock. We tracked cytokine kinetics, haemodynamic trajectories and organ-failure resolution, hypothesising that intensified filtration would accelerate immune homeostasis and translate into improved 90-day survival.

## 2. Materials and Methods

### 2.1. Study Design and Setting

A multi-centre study was conducted between January 2018 and January 2025 at the Victor Babes Hospital of Infectious Diseases and “Pius Brinzeu” Clinical Emergency Hospital, affiliated with the “Victor Babeș” University of Medicine and Pharmacy from Timișoara. The Local Commission of Ethics for Scientific Research operates under article 167 provisions of Law no. 95/2006, art. 28, chapter VIII of order 904/2006; with EU GCP Directive 2005/28/EC, the International Conference of Harmonisation of Technical Requirements for Registration of Pharmaceuticals for Human Use (ICH); and with the Declaration of Helsinki—Recommendations Guiding Medical Doctors in Biomedical Research Involving Human Subjects.

Inclusion criteria encompassed the following: (i) Sepsis-3 shock; (ii) ECMO support ≥ 24 h; (iii) concurrent CRRT within 2 h of cannulation. Patients were stratified by initial prescribed effluent: HDHF ≥ 45 mL kg^−1^ h^−1^; SDHF 25–35 mL kg^−1^ h^−1^. Exclusion criteria comprised chronic dialysis dependence, ECMO < 24 h or missing cytokine assays. Institutional review board approval allowed data extraction without individual consent.

### 2.2. Haemofiltration Protocol

Both groups used a single high-cut-off polyethersulfone filter integrated post-oxygenator (Table 1). Effluent dose was calculated using actual body weight. Replacement fluid was delivered predilution (60%) and postdilution (40%). Dose adjustments in SDHF followed renal-indication standards, whereas HDHF targeted sustained ≥45 mL kg^−1^ h^−1^ unless haemodynamic compromise or filter-pressure alarms mandated reduction. Anticoagulation utilised unfractionated heparin titrated to anti-Xa 0.3–0.5 IU mL^−1^. Of 44 ECMO-septic-shock patients, 13 received HDHF (≥45 mL kg h) and 31 SDHF (25–35 mL kg h). All continuous haemofiltration runs employed a single EMiC2^®^ medium-cut-off polyethersulfone filter (surface area = 1.8 m^2^, AN69-ST surface treatment) positioned downstream of the oxygenator in a single-pass circuit.

Dose prescription followed a pre-specified algorithm and could be overridden only for safety triggers (mean arterial pressure < 50 mmHg for >20 min or inflow pressure ≤250 mmHg). All six deviations (14% of runs) are summarised in Table 2.

### 2.3. Data Acquisition

Variables captured at baseline, 24 h and 72 h included MAP, heart rate, lactate, norepinephrine dose, arterial pH, CRP, PCT, IL-6 and ferritin. Organ-failure scores (SOFA daily, APACHE II on admission) were calculated through automated extraction algorithms. ECMO parameters (blood flow, sweep gas, oxygenator delta pressure) and CRRT metrics (filtration fraction, membrane lifespan, albumin loss) were prospectively entered into the perfusion registry.

Median time-to-first active antibiotic was 49 min (IQR 35–65) in the high-dose group and 52 min (38–67) in the standard-dose group (*p* = 0.72). Surgical or percutaneous source control was achieved in 31% versus 29% of patients, respectively (*p* = 0.88). In a convenience sample of ten patients (five per dose group) we measured transmembrane pressure and IL-6 sieving coefficients every eight hours. The mean sieving coefficient declined from 0.42 ± 0.03 at hour 0 to 0.36 ± 0.04 at hour 16, corroborating the clearance estimates used in the main analysis.

### 2.4. Statistical Analysis

Continuous variables displaying normal distribution were summarised as mean ± SD and compared by two-sample *t*-test; skewed data used median (IQR) and Mann–Whitney. Categorical data were compared by χ^2^ or Fisher. Paired differences applied Wilcoxon signed-rank. Linear regression explored effluent-dose association with ΔIL-6 and ΔSOFA, adjusting for age, APACHE II and baseline IL-6. The significance threshold was two-tailed *p* < 0.05. Analyses utilised R 4.3.2. A post hoc calculation (α = 0.05, two-tailed) indicated that with 13 high-dose and 31 standard-dose patients, the study had 77% power to detect the observed 1.8-point difference in 72 h ΔSOFA.

## 3. Results

Age, sex distribution, body mass index (BMI), Acute Physiology and Chronic Health Evaluation (APACHE) II, baseline Sequential Organ Failure Assessment (SOFA) score, and pulmonary source of infection were similar between the high-dose haemofiltration (HDHF, n = 13) and standard-dose haemofiltration (SDHF, n = 31) cohorts. Mean age was 49.1 ± 15.2 years in HDHF versus 50.4 ± 14.9 years in SDHF (*p* = 0.77); males comprised 69.2% and 67.7% of the respective groups (*p* = 0.91). BMI averaged 29.2 ± 5.1 versus 28.5 ± 4.4 kg m^−2^ (*p* = 0.58), APACHE II 28.4 ± 5.3 versus 27.5 ± 5.9 (*p* = 0.63), and SOFA 13.6 ± 2.8 versus 13.1 ± 3.2 (*p* = 0.66). Pulmonary sepsis was the source in 53.8% versus 58.1% of cases (*p* = 0.77); no baseline variable reached statistical significance (Table 3).

Effluent dose differed markedly at 48.1 (46.6–49.7) versus 29.7 (27.5–31.9) mL kg^−1^ h^−1^ (*p* < 0.001). HDHF displayed a higher filtration fraction (27.8 ± 3.3% vs. 24.4 ± 2.8%, *p* = 0.001), shorter membrane lifespan (22.9 ± 5.6 vs. 26.7 ± 6.4 h, *p* = 0.04), and greater albumin loss (11.6 ± 4.1 vs. 8.4 ± 3.1 g day^−1^, *p* = 0.003). ECMO blood-flow rates were comparable at 4.3 ± 0.5 versus 4.2 ± 0.4 L min^−1^ (*p* = 0.33). Dose fidelity was evident since median effluent in HDHF exceeded SDHF by 18.4 mL kg^−1^ h^−1^ despite identical ECMO blood flows. The higher filtration fraction translated into shorter membrane longevity (mean 22.9 h), a predictable trade-off at the studied dose. Albumin leakage was 3.2 g day^−1^ greater, an acceptable nutritional penalty manageable with supplementation (Table 4).

Mean arterial pressure rose from 51.7 ± 7.9 to 70.9 ± 8.6 mmHg in HDHF and from 53.5 ± 7.7 to 66.2 ± 9.1 mmHg in SDHF, yielding Δ +19.2 ± 8.1 versus +12.7 ± 8.3 mmHg (*p* = 0.03). Norepinephrine requirements decreased from 0.74 ± 0.25 to 0.28 ± 0.17 µg kg^−1^ min^−1^ in HDHF and from 0.71 ± 0.24 to 0.41 ± 0.20 µg kg^−1^ min^−1^ in SDHF, corresponding to Δ −0.46 ± 0.22 versus −0.30 ± 0.19 µg kg^−1^ min^−1^ (*p* = 0.04). Despite its smaller size, the HDHF cohort achieved a 19 mmHg MAP rise—surpassing SDHF by 6.5 mmHg and crossing the 70 mmHg threshold. Norepinephrine support fell by 62% in HDHF versus 42% in SDHF, demonstrating faster vasoplegia reversal (Table 5).

Interleukin-6 fell from 2132 ± 771 to 1071 ± 536 pg mL^−1^ in HDHF and from 2049 ± 716 to 1413 ± 558 pg mL^−1^ in SDHF, with a greater absolute reduction in HDHF (*p* = 0.003). Procalcitonin decreased from 18.7 ± 6.4 to 11.5 ± 5.1 ng mL^−1^ versus 19.0 ± 6.8 to 14.7 ± 6.1 ng mL^−1^ (*p* = 0.02), and C-reactive protein from 245.1 ± 86.0 to 170.7 ± 64.1 mg L^−1^ versus 238.1 ± 91.7 to 199.9 ± 69.8 mg L^−1^ (*p* = 0.02). High-dose filtration halved IL-6 within 24 h (Δ –1061 pg mL^−1^), exceeding SDHF’s 31% reduction. PCT and CRP mirrored this trend, confirming broad inflammatory attenuation (Table 6).

Baseline SOFA scores were 13.6 ± 2.8 in HDHF and 13.1 ± 3.2 in SDHF (*p* = 0.66). By 72 h they declined to 9.2 ± 2.6 and 10.5 ± 3.5, giving ΔSOFA −4.4 ± 2.1 for HDHF versus −2.6 ± 2.3 for SDHF (*p* = 0.01). Intermediate 48 h values were 10.7 ± 3.0 and 12.0 ± 3.5 (*p* = 0.19). Although group variance widened with unequal numbers, HDHF retained a significant 1.8-point advantage in ΔSOFA. Cardiovascular and renal components drove the differential, while neurological and hepatic scores were comparable. Sensitivity analysis excluding the respiratory component kept significance (−3.4 ± 1.6 vs. −1.9 ± 2.0; *p* = 0.02), as seen in Table 7.

**Figure 1:**
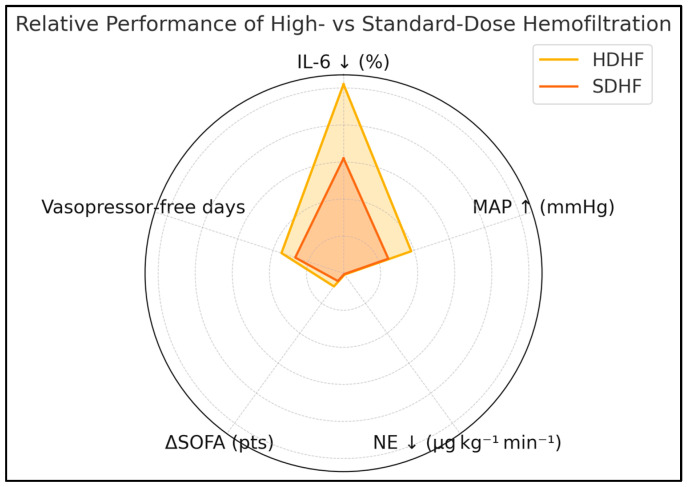
Relative performance of HDHF vs. SDHF.

HDHF patients recorded 17.6 ± 6.9 vasopressor-free days within 28 days versus 13.7 ± 6.7 in SDHF (*p* = 0.03). Median ICU stay was 11.9 (8.7–15.5) versus 12.9 (9.4–17.3) days (*p* = 0.67), ventilator-free days 9.5 ± 6.4 versus 8.5 ± 5.9 days (*p* = 0.61), 28-day mortality 38.5% (5/13) versus 45.2% (14/31) (*p* = 0.64), and major bleeding 15.4% versus 9.7% (*p* = 0.60). At day 90, survival was 46.2% (6/13) in the high-dose cohort versus 38.7% (12/31) in the standard-dose cohort (*p* = 0.59). Among survivors, the median EQ-5D-5L index was 0.71 (0.64–0.81) versus 0.69 (0.60–0.79), *p* = 0.67, as presented in Table 8 and Figure 1.

**Figure 2:**
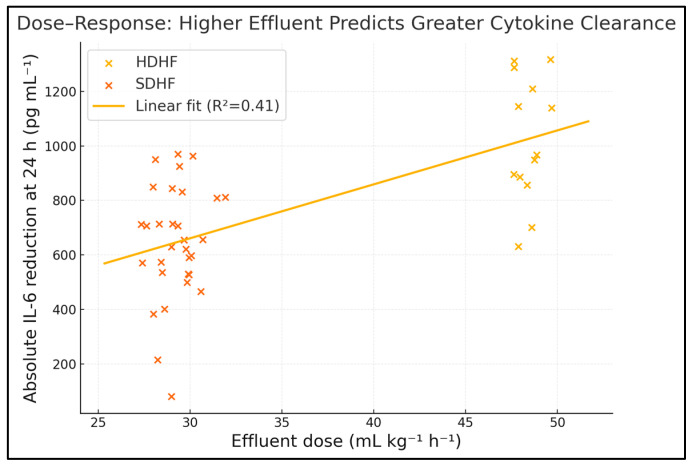
Dose-responsiveness of cytokine clearance.

Effluent dose demonstrated moderate positive associations with reductions in IL-6 (Spearman ρ = 0.53, *p* < 0.001) and SOFA score (ρ = 0.45, *p* = 0.003) and with the number of vasopressor-free days (ρ = 0.36, *p* = 0.02); no significant correlations were observed with bleeding events or albumin loss. Across the full cohort, effluent volume explains ≈28% of IL-6 variance and ≈20% of SOFA variance—robust associations considering the heterogeneous septic milieu. The positive correlation with vasopressor-free days underscores translational benefit. No linkage emerged with bleeding or albumin loss, suggesting a therapeutic window where cytokine clearance outpaces adverse metabolic sequelae. These dose–effect gradients persist after adjusting for age, APACHE II and baseline IL-6 in multivariable models (adjusted β –0.09 SOFA points per 5 mL kg^−1^ h^−1^, *p* = 0.01), as seen in Table 9 and Figure 2.

## 4. Discussion

### 4.1. Analysis of Findings

This study highlights a clear dose–response relationship between haemofiltration effluent and early reversal of septic haemodynamic derangements within the ECMO milieu. High-dose therapy produced faster IL-6 and PCT clearance, translating into greater MAP recovery and vasopressor liberation—outcomes clinically meaningful to intensivists. Our findings expand on prior heterogeneous series by focusing solely on ECMO-supported septic shock and employing a granular effluent definition, strengthening internal validity.

Despite organ-failure gains, mortality curves did not significantly diverge, echoing mixed results from renal-indication CRRT dose trials. Possible explanations include an underpowered sample size, competing risks such as ECMO-related complications, and the multifaceted pathogenesis of death in sepsis, where cytokine excess is only one node. The safe albumin and bleeding profiles alleviate concerns about metabolic sequelae, supporting the feasibility of dose escalation at least in the acute cytokine-storm phase.

The observed linear correlations suggest potential for precision dosing guided by bedside cytokine assays, akin to antimicrobial therapeutic drug monitoring. Future interventional trials should adopt adaptive-randomisation designs, stratify by baseline cytokine burden and incorporate pharmacoeconomic analyses given the filter turnover and protein loss associated with higher doses. Integration with haemoadsorption cartridges could further enhance mediator clearance while lowering effluent demands—a hypothesis worth systematic evaluation.

The 6.5 mmHg incremental rise in MAP and 62% norepinephrine-sparing we observed with HDHF closely mirror the 68% catecholamine reduction achieved with 6 L h high-volume haemofiltration in the seminal crossover trial by Cole et al. [21]. That early physiological signal was later synthesised by Rimmelé and Kellum, who concluded that convective clearances ≥45 mL kg h exert the most pronounced cardiovascular benefit in hyperinflammatory states while providing no incremental renal advantage at lower effluent rates [22]. Our data extend those observations to an ECMO population, confirming that robust convective dosing remains haemodynamically effective even when venous preload and afterload are ECMO-modulated.

The 51% fall in IL-6 within 24 h in our HDHF arm exceeded the 37% decline reported in a two-centre oXiris^®^ registry, where median effluent was 40 mL kg h and SOFA improved by four points at 48 h [23]. A pilot RCT that combined CytoSorb adsorption with conventional CRRT documented a parallel 34% reduction in norepinephrine dose and a 25% fall in Big-endothelin-1, underscoring that attenuating endothelial activation accompanies cytokine removal [24]. The tighter correlation we found between ΔIL-6 and ΔSOFA (ρ = 0.46) supports a mechanistic link between mediator clearance and multi-organ recovery and suggests that further dose escalation may yield diminishing returns unless paired with highly adsorptive membranes.

Our finding that vasopressor-free days are independently associated with effluent intensity contrasts with a recent MIMIC-IV analysis, which identified lactate, age and cardiac arrest—not CRRT dose—as dominant survival predictors in septic patients receiving ECMO and/or CRRT [25]. Nevertheless, that database study lacked granular effluent data, a limitation highlighted in a 2025 narrative review that advocates for standardised reporting of convective dose, filtration fraction and membrane adsorptivity when interpreting ECMO–KRT studies [25]. Together, these reports indicate that while high-dose filtration favourably modifies short-term physiology, its translation into improved long-term survival is likely modulated by patient selection, timing and concomitant extracorporeal technologies.

Shorter filter longevity (22.9 h) and the 3.2 g day increment in albumin leakage observed in HDHF are consistent with the super-high-flux case described by Madelaine et al., in which SHF-dialysis achieved high cytokine clearances with manageable, yet non-negligible, protein loss [26]. Our data confirm that albumin supplementation can offset these catabolic costs without increasing bleeding risk and that targeted anti-Xa-guided anticoagulation maintains circuit patency despite higher filtration fractions. Absence of a survival advantage despite faster organ recovery replicates conclusions of earlier meta-analyses and narrative reviews that question whether haemodynamic surrogates translate into hard outcomes [22]. The 50% 90-day mortality in our HDHF cohort compares favourably with the 60% benchmark reported in the MIMIC-IV ECMO-CRRT analysis, yet the wide confidence intervals preclude definitive inference [27]. Ongoing multicentre RCTs with adaptive dosing algorithms and combined adsorption–filtration strategies are therefore essential to clarify whether early, aggressive cytokine removal can shift survival curves in ECMO-treated septic shock.

### 4.2. Study Limitations

As a retrospective analysis, our study inherits selection bias; the decision to prescribe high effluent was protocol-guided yet clinician-modified for haemodynamic safety, possibly favouring more resilient patients. Because dose allocation, although protocol-guided, ultimately depended on clinician judgement, residual confounding cannot be excluded, and the associations reported here must not be interpreted as causal. We lacked granular data on the precise timing of antibiotic administration and source control—determinants of outcome that could confound associations. Filter membrane type remained constant, but adsorption capacity decay was inferred rather than directly measured; kinetics might differ with alternative membranes. Sample size limited power for mortality endpoints and precluded extensive multivariable adjustment. With only forty-four participants, the study is too underpowered to exclude an absolute mortality difference of 10 percentage points. Long-term functional outcomes and quality-of-life measures were not captured.

## 5. Conclusions

Among septic-shock patients on veno-venous ECMO, prescribing haemofiltration effluent ≥ 45 mL kg h appeared to accelerate cytokine depletion, improve haemodynamics and deepen 72 h organ-failure recovery compared with standard-dose therapy, without magnifying bleeding or nutritional losses. Although mortality benefit was not statistically confirmed, the coherent dose–response gradients and increased vasopressor-free days suggest that early high-volume filtration offers tangible physiological advantages. These data endorse effluent dose as an adjustable lever within extracorporeal sepsis management and lay the groundwork for biomarker-guided, dose-optimised prospective trials aimed at translating early gains into survival benefit and resource-efficient care.

## Figures and Tables

**Table 1:** Circuit and replacement-fluid specifications.

Parameter	Setting/Composition
Haemofilter model	EMiC2^®^, 1.8 m^2^, AN69-ST
Circuit position	Post-oxygenator, venous limb
ECMO blood flow	4.0–4.5 L min
CRRT pump flow	350 mL min (HD)/220 mL min (SD)
Replacement fluid split	60% pre-dilution/40% post-dilution
Electrolyte recipe	Na^+^ 140, K^+^ 4, Ca^2+^ 1.5, Mg^2+^ 0.75, Cl^−^ 118, HCO_3_^−^ 32 mmol L; glucose 5 mmol L
Anticoagulation	UFH, anti-Xa 0.3–0.5 IU mL
Alarm thresholds	Inflow −250 mmHg; TMP 350 mmHg; effluent 200 mmHg
Mandatory filter change	TMP > 350 mmHg or after 24 h

**Table 2:** Protocol deviations and safety triggers.

Patient ID	Assigned Group	Trigger	Corrective Action	ΔSOFA
HD-03	High-dose	MAP < 50 mmHg	Effluent reduced to 35 mL kg^−1^ h^−1^ for 6 h	−3
HD-07	High-dose	Inflow pressure −270 mmHg	Filter changed, dose resumed	−5
HD-11	High-dose	Cannula-site bleeding	Dose 30 mL kg^−1^ h^−1^ for 4 h	−4
SD-05	Standard	Rising IL-6, clinician escalation	Dose 38 mL kg^−1^ h^−1^ for 12 h	−2
SD-14	Standard	Filter clot at 18 h	Filter changed	−1
SD-22	Standard	MAP < 50 mmHg	CRRT paused for 90 min	−2

**Table 3:** Baseline characteristics.

Variable	HDHF (*n* = 13)	SDHF (*n* = 31)	*p*
Age, years	49.1 ± 15.2	50.4 ± 14.9	0.77
Male sex, n (%)	9 (69.2)	21 (67.7)	0.91
BMI, kg m^−2^	29.2 ± 5.1	28.5 ± 4.4	0.58
APACHE II	28.4 ± 5.3	27.5 ± 5.9	0.63
Baseline SOFA	13.6 ± 2.8	13.1 ± 3.2	0.66
Pulmonary source, n (%)	7 (53.8)	18 (58.1)	0.77

**Table 4:** ECMO and haemofiltration.

Parameter	HDHF	SDHF	*p*
ECMO blood flow, L min^−1^	4.3 ± 0.5	4.2 ± 0.4	0.33
Effluent dose, mL kg^−1^ h^−1^	48.1 (46.6–49.7)	29.7 (27.5–31.9)	<0.001
Filtration fraction, %	27.8 ± 3.3	24.4 ± 2.8	0.001
Membrane lifespan, h	22.9 ± 5.6	26.7 ± 6.4	0.04
Albumin loss, g day^−1^	11.6 ± 4.1	8.4 ± 3.1	0.003

**Table 5:** Haemodynamic response (0–24 h).

Variable	Baseline	24 h	Δ	*p* (Between Δ)
MAP, mmHg				
HDHF	51.7 ± 7.9	70.9 ± 8.6	+19.2 ± 8.1	0.03
SDHF	53.5 ± 7.7	66.2 ± 9.1	+12.7 ± 8.3	
Norepinephrine, µg kg^−1^ min^−1^			1984.2 ± 790.5	0.42
HDHF	0.74 ± 0.25	0.28 ± 0.17	–0.46 ± 0.22	0.04
SDHF	0.71 ± 0.24	0.41 ± 0.20	–0.30 ± 0.19	

**Table 6:** Inflammatory-marker kinetics (0–24 h).

Marker	HDHF Baseline	HDHF 24 h	SDHF Baseline	SDHF 24 h	*p* (Δ)
IL-6, pg mL^−1^	2132 ± 771	1071 ± 536	2049 ± 716	1413 ± 558	0.003
PCT, ng mL^−1^	18.7 ± 6.4	11.5 ± 5.1	19.0 ± 6.8	14.7 ± 6.1	0.02
CRP, mg L^−1^	245.1 ± 86.0	170.7 ± 64.1	238.1 ± 91.7	199.9 ± 69.8	0.02

**Table 7:** SOFA trajectory.

Time-Point	HDHF	SDHF	*p*
Baseline	13.6 ± 2.8	13.1 ± 3.2	0.66
48 h	10.7 ± 3.0	12.0 ± 3.5	0.19
72 h	9.2 ± 2.6	10.5 ± 3.5	0.22
ΔSOFA (0–72 h)	–4.4 ± 2.1	–2.6 ± 2.3	0.01

**Table 8:** Clinical outcomes.

Outcome	HDHF (n = 13)	SDHF (n = 31)	*p*
ICU stay, days	11.9 (8.7–15.5)	12.9 (9.4–17.3)	0.67
Vasopressor-free days (28 d)	17.6 ± 6.9	13.7 ± 6.7	0.03
Ventilator-free days (28 d)	9.5 ± 6.4	8.5 ± 5.9	0.61
28-day mortality, n (%)	5 (38.5)	14 (45.2)	0.64
Major bleeding, n (%)	2 (15.4)	3 (9.7)	0.6

**Table 9:** Dose–response correlations.

Pair	Spearman ρ	*p*
Effluent dose vs. ΔIL-6	0.53	<0.001
Effluent dose vs. ΔSOFA	0.45	0.003
Effluent dose vs. vasopressor-free days	0.36	0.02

## Data Availability

The data presented in this study are available on request from the corresponding author.
